# Epistasis Activation Contributes Substantially to Heterosis in Temperate by Tropical Maize Hybrids

**DOI:** 10.3389/fpls.2022.921608

**Published:** 2022-07-11

**Authors:** Zhiqin Sang, Hui Wang, Yuxin Yang, Zhanqin Zhang, Xiaogang Liu, Zhiwei Li, Yunbi Xu

**Affiliations:** ^1^Institute of Crop Sciences, Chinese Academy of Agricultural Sciences, Beijing, China; ^2^Xinjiang Academy of Agricultural and Reclamation Science, Shihezi, China; ^3^Crop Research Institute, Shandong Academy of Agricultural Sciences, Jinan, China; ^4^National Engineering Research Center of Wheat and Maize, Shandong Technology Innovation Center of Wheat, Shandong Academy of Agricultural Sciences, Jinan, China; ^5^International Maize and Wheat Improvement Center, Texcoco, Mexico

**Keywords:** maize, heterosis, GWAS, epistatic effects, protein–protein interaction, multiple-hybrid population

## Abstract

Epistasis strongly affects the performance of superior maize hybrids. In this study, a multiple-hybrid population, consisting of three hybrid maize sets with varied interparental divergence, was generated by crossing 28 temperate and 23 tropical inbred lines with diverse genetic backgrounds. We obtained 1,154 tested hybrids. Among these tested hybrids, heterosis increased steadily as the heterotic genetic distance increased. Mid-parent heterosis was significantly higher in the temperate by tropical hybrids than in the temperate by temperate hybrids. Genome-wide prediction and association mapping was performed for grain weight per plant (GWPP) and days to silking (DTS) using 20K high-quality SNPs, showing that epistatic effects played a more prominent role than dominance effects in temperate by tropical maize hybrids. A total of 33 and 420 epistatic QTL were identified for GWPP and DTS, respectively, in the temperate by tropical hybrids. Protein–protein interaction network and gene-set enrichment analyses showed that epistatic genes were involved in protein interactions, which play an important role in photosynthesis, biological transcription pathways, and protein synthesis. We showed that the interaction of many minor-effect genes in the hybrids could activate the transcription activators of epistatic genes, resulting in a cascade of amplified yield heterosis. The multiple-hybrid population design enhanced our understanding of heterosis in maize, providing an insight into the acceleration of hybrid maize breeding by activating epistatic effects.

## Introduction

Hybrid vigor or heterosis refers to the phenotypic superiority of F_1_ hybrid plants over their parents. The mechanism of heterosis can be determined according to three primary hypotheses based on classical genetics. The dominance hypothesis explains heterosis by the action of superior dominant alleles from both parents at multiple loci, which complement corresponding unfavorable alleles leading to the enhancement of hybrid vigor. Such complementation might allow hybrids to be similar to or better than the superior parent (Jones, 1917; Birchler, 2015). According to the single locus overdominance hypothesis, various alleles interact to perform a function better than that performed by homozygous alleles. Therefore, the increase in vigor is proportional to the degree of heterozygosity (East, 1936; Lamkey and Edwards, 1999). Dominance and overdominance hypotheses are based on the action of a single gene, but most heterosis-related traits are quantitatively inherited and involve multiple genes with different effects. The epistasis hypothesis emphasizes the role of inter-allele interactions among genetic loci and associated pathways, which might include all possible forms of molecular interactions (Powers, 1944; Jinks and Jones, 1958; Jiang et al., 2017). Many researchers suggested that partial or complete dominance, rather than super dominance, account for the inheritance of heterosis in maize (Hallauer et al., 2010). Although some researchers have focused on single-gene models, several studies have suggested that heterosis is generally the result of the action of multiple loci, which affect heterosis of different traits and hybrids (Bauman, 1959; Doebley et al., 1995; Ma et al., 2007; Hallauer et al., 2010).

As East (1936) stated, “the problem of heterosis is the problem of the inheritance of quantitative characters,” and quantitative traits are typically affected by multiple genes (Flint-Garcia et al., 2009). The genotype of a population has a “net-like” structure; hence, different loci might affect the variation in several characters (Wright, 1984). Additionally, substituting one gene might affect several characters (Yu et al., 1997; Xiao et al., 2021). Based on this perspective, epistasis is one of the most important genetic components in the inheritance of quantitative characters. Epistasis might also contribute to the genetic basis of heterosis (Hua et al., 2003; Hochholdinger and Baldauf, 2018). Epistasis not only shapes which loci can express heterosis but can also mimic overdominance (Fiévet et al., 2010). Several studies have investigated the epistatic effects using quantitative and biparental population genetic approaches (Doebley et al., 1995; Culverhouse et al., 2004). Biparental populations have a narrow genetic basis, which further restricts the effective detection of epistasis (Jiang et al., 2017; Xiao et al., 2021). Due to the lack of tailored quantitative genetic approaches to determine the function of epistasis in hybrid breeding populations, epistatic effects in the populations developed by crossing a panel of diverse breeding materials could not be assessed (Boeven et al., 2020). A quantitative genetic framework was developed to determine the relative contributions of dominance and epistatic effects to heterosis (Jiang et al., 2017), allowing the integration of epistasis in hybrid populations to derive from different parents.

In line with the quantitative genetic hypothesis, hybrid vigor is determined by the interparental genetic distance (Wei and Zhang, 2018). Assuming that all quantitative trait loci (QTL) contribute to heterosis, the genetic distance can be estimated by the squared difference of the interparental allele frequency (Frankham, 1996; Boeven et al., 2020). When heterosis mainly results from dominance and overdominance effects, it is positively correlated with genetic distance (Lamkey and Edwards, 1998). The genetic distance between parents in maize was found to be moderately or highly correlated with middle parent heterosis (Laude and Carena, 2015), which was contrary to several other reports from the tropical region (Badu-Apraku et al., 2013; Oyekunle et al., 2015). Most artificial selection techniques involved reshaping gene networks rather than single genes (Doust et al., 2014). Thus, due to thousands of years of artificial and natural selection, temperate germplasm exhibits significantly lower genetic diversity compared to tropical germplasm, as shown by the diversity of haplotypes and SNP markers (Lu et al., 2009, 2011). Therefore, the genetic distance between temperate and tropical maize is greater than that within temperate maize. Ignoring the genetic architecture of heterosis within and between alleles might show incorrect relationships between heterosis and genetic distance, especially for the temperate by tropical hybrids with significant genetic differentiation (Boeven et al., 2020).

To understand the genetic mechanism of heterosis in different types of maize, three different hybrid maize panels were developed in this study. The temperate by temperate panel comprised 377 temperate maize hybrids, the temperate by tropical panel comprised 641 temperate and tropical hybrids, and the tropical by tropical panel comprised 136 tropical hybrids. In this study, we found that the heterosis of grain yield and flowering stage increased with heterotic genetic distance. Genome-wide association studies on the heterosis of grain yield and days to silking revealed changes in the genetic architecture of the hybrids from the temperate by temperate to temperate by tropical panels, indicating novel epistatic effects underlying the heterosis in the temperate by tropical panel. The results of the protein–protein interaction (PPI) network analysis and gene-set enrichment analysis (GSEA) demonstrated that many minor loci interacting with the gene networks might improve the performance of the hybrids.

## Materials and Methods

### Plant Materials and Phenotyping

The multiple-hybrid population used in this study was derived from the cross between 23 tropical and 28 temperate inbred lines, representing high diversity in temperate and tropical regions. These lines were grouped into seven heterotic pools according to their pedigrees, original regions, and genetic population structure inferred from molecular markers (Wang et al., 2017). The temperate diallel contained 377 hybrids derived from Griffing IV (temperate by temperate) with 28 temperate maize inbred lines (13 U.S. and 15 Chinese lines), representing different heterotic groups. The NC II (temperate by tropical) hybrid panel contained 641 hybrids generated from 23 tropical and 28 temperate inbred lines. The tropical diallel contained 136 hybrids derived from Griffing IV (tropical by tropical) with 17 tropical inbred lines as parents, many of which were developed at the International Maize and Wheat Improvement Center (CIMMYT) (Supplementary Figure 1). For 325 temperate diallel crosses and 263 NC II crosses, enough hybrid seeds were generated to conduct field trials for phenotyping in 2013, 2014, and 2015 at Xinxiang, Henan (35.1°N, 113.8°E) and Shunyi, Beijing (40.2°N, 116.6°E) using randomized block design with two duplicates for each experiment (Wang et al., 2017; Yu et al., 2020). Tropical diallel hybrids were phenotyped in Jinghong, Yunnan (22.0°N, 100.8°E) in 2014 and Sanya, Hainan (18.4°N, 109.2°E) in 2015 using the same experimental design (Wang et al., 2017). For 377 temperate diallel crosses and 641 NC II crosses, enough hybrid seeds were generated to conduct field trials for 2 years (2017–2018) in Shihezi, Xinjiang (45.2°N, 84.68°E), using alpha designs with two replicates (Supplementary Table 1). For each replicate, during field trials, all the hybrids and their 51 parents were split into two adjacent trials. Herbicides, insecticides, and fertilizers were applied following the farmer’s practices in intensive maize production.

Days to silking (DTS) and days to anthesis (DTA) were recorded as the number of days from planting to when 50% of the plants in a plot had shed pollen and extruded silks, respectively; anthesis–silking interval (ASI) was defined as the time interval between DTS and DTA. After female flowering, the PH values were recorded as the averaged value of the five plants from the center of the plot. The grain yield per plant (GWPP) was estimated based on the mean value of 10 plants. The grain number per row (GNPR), the row number (RN), and the ear barren tips (TIP) were measured as the averaged value of 10 ears from each plot. Harvesting was performed manually, and the harvest was adjusted to a moisture content of 140 g H_2_O kg^–1^.

### Phenotypic Data Analysis

A two-stage method was adopted for phenotypic data analysis (Möhring and Piepho, 2009), where effects were modeled and estimated for each environment first, and then the means of genotypes across environments were calculated. In the first step, we used a mixed model approach for analyzing the modeling effects of individual environments for genotypes, replications, and the blocks within replications, based on the statistical model


(1) $$y_{i\,j\,k\,l}=g_{i\,j}+r_{k}+b_{l\,k}+e_{i\,j\,k\,l}$$

where *y*_*ijk*_ represents the phenotypic performance of the *ij*th genotype (hybrid *j*≠*i* or parental line *i* = *j*) within the *l*th incomplete block for the *k*th replication, *g*_*ij*_ represents the genetic effect of the *ij*th genotype, *r*_*k*_ represents the effect of the *k*th replication, *b*_*lk*_ represents the effect of the *l*th incomplete block within the *k*th replication, and *e*_*ijkl*_ represents the residual. Except for the effect of *g*_*ij*_, the remaining effects were random.

In the second step, to analyze the phenotypes in various environments, we used a linear mixed model based on the adjusted best linear unbiased estimates (BLUEs) of individual environments:


(2) $$y_{i\,j\,n}=g_{i\,j}+l_{n}+\left(g_{i\,j}\,l\right)+e_{i\,j\,n}$$

where *g*_*ij*_ and *l*_*n*_ denote the genotypes and individual environments, respectively. Except for the effect of *g*_*ij*_, all other effects were random.

The fixed genotypic effects were used to obtain the BLUEs for parental and hybrid genotypic values. Next, we used the BLUEs for calculating mid-parent heterosis (_*MPH*_) of all hybrids by *MPH* = *F*_1_−*MP*, where, *F*_1_ represents the hybrid performance, and *MP* represents the mid-parent value for two parents, *P*_1_ and *P*_2_. Then, we determined the relative *MPH*(%) for all hybrids using the formula $MPH\left(\%\right)=\left(\frac{M\,P\,H}{M\,P}\right)\times 100$. Next, we determined better-parent heterosis (_*BPH*_) using the formula *BPH* = *F*_1_−*P*_Better_, where *P*_Better_ represents the performance of the better-performing parental line. We measured relative *BPH* using the formula $BPH\left(\%\right)=\left(\frac{B\,P\,H}{P_{\text{Better}}}\right)\times 100$. Pearson’s product–moment correlation coefficients were calculated to test the BLUEs-based correlations. Student’s *t*-tests were performed to compare the BLUEs among diverse genotypes.

The mixed linear model approach was used to estimate variance components, and except for the group effect, the remaining effects were random. Then, we decomposed the total variance of the temperate by temperate and temperate by tropical set to the variances resulting from the effects of the general combining ability (GCA) of female and male subjects, as well as the variance resulting from the specific combining ability (SCA) of the hybrids (Zhao et al., 2015):


(3) $$y_{i\,j\,k\,l}=a+l_{n}+l\,r_{n\,k}+g_{i}+g_{j}+g_{i\,j}+g_{i}:l_{n}+g_{j}:l_{n}+g_{i\,j}:l_{n}+e_{i\,j\,n}$$

where *y*_*ijkl*_ represents the phenotypic performance of the *ij*th entry (hybrid *i*≠*j*, or line *i* = *j*) in the *n*th environment, *a* represents hybrid and line group effects, *l*_*n*_ indicates the effect of the *n*th environment, *g*_*i*_ and *g*_*j*_ indicate the genetic effects of the parental lines, *g*_*ij*_ represents the SCA effect of the crosses between *i* and *j* lines, *g*_*i*_:*l*_*n*_ and *g*_*j*_:*l*_*n*_ represents the interplay effect between the *i*/*j*th parental lines and the *n*th environment, *g*_*ij*_:*l*_*n*_ represents the interplay effect between the SCA and the environment, whereas, *e*_*ijn*_ represents the residual. Heritability was estimated by the genotypic-to-phenotypic variance ratio,$\,H^{2}=\frac{\delta_{G}^{2}}{\delta_{G}^{2}+\delta_{G}^{2}/E+\delta_{e}^{2}/\left(E\times R\right)}$, where, *E* represents environment number, *R* represents the mean replication number in each entry in one location, and $\delta_{e}^{2}$ represents the combined error variance. The MPH was estimated according to the block-corrected values of the hybrids in each environment and their parental BLUEs obtained across all environments. These MPH values calculated for each environment were used to construct the linear mixed model (Equation 3) and estimate heritability. The ASReml-R 4.0 software package was used for performing statistical analysis in the R environment (Butler et al., 2009).

### Genotypic Data Analysis

In a previous study, we genotyped the 51 parental lines with the Maize55K chip (Wang et al., 2017; Xu et al., 2017). From this SNP dataset, quality control of minor allele frequency (MAF>5%), missing data (<5%), and SNP were filtered based on linkage disequilibrium (LD) (eliminating one in each SNP pair when LD was greater than 0.5 among 50 SNPs). We retained 22,510 high-quality SNP markers for further analyses. For some SNPs with less than 5% MAF in the temperate hybrids, 20,555 high-quality SNPs were finally selected and used for genome-wide prediction and association mapping in the temperate by temperate set. The marker profiles for each hybrid were deduced from the corresponding parental lines. In the genome-wide prediction and association mapping of the additive model, the minor homozygote was coded as “2,” the major homozygote was coded as “0,” and the heterozygote was coded as “1,” The *F*_*st*_ statistic and SNP nucleotide divergence (π) between the temperate and tropical inbred lines were calculated using VCFtools (Danecek et al., 2011) and visualized in R (R Core Team, 2018). For each 1,000-kb window, we calculated the *F*_*st*_ statistics and sequence diversity statistics (π) with 100-kb steps in the maize genome. We also determined π values for temperate and tropical inbred lines, and the ratio of π (π_temperate_/π_tropical_) was used to detect the genetic-improvement sweeps. For parental lines and hybrids, the LD decomposition, based on the genetic map distance, was evaluated by fitting the natural smoothing splines to *r*^2^ values using the software package PLINK (Purcell et al., 2007). Principal component analysis (PCA) was performed to analyze the population structures of the hybrids and parental lines using the software TASSEL (Bradbury et al., 2007), and MEGA version 7.0.26 was used for clustering based on modified Nei’s genetic distance (Tamura et al., 2013). Rogers’ distance (RD) was used to measure the genetic distance (Boggs and Rogers, 1990). We conducted genome-wide prediction for the hybrid phenotype to obtain dominance effects for GWPP and DTS (Zhao et al., 2013; Alves et al., 2019); then, we used them to weigh the marker loci. Furthermore, we determined the heterotic genetic distance developed by Boeven et al. (2020), which was expressed depending on Rogers’ distance by including the predicted dominance effects for SNP, as shown in Equation 4:


(4) $$\int_{RD}(X,Y)=\frac{1}{L}\sum_{u=1}^{L}\sqrt[{w_{u}}]{\frac{\sum_{j=1}^{n_{u}}{\left(X_{uj-}Y_{uj}\right)}^{2}}{2}}$$

where *X* and *Y* represent two genotypes, *X*_*uj*_ and *Y*_*uj*_ represent the frequency of the *j*th allele at the *u*th locus, *n*_*u*_ represents the allele number in the *u*th locus, *L* represents the locus number, and *w*_*u*_ indicates the dominance weight at the_  u_th locus. Bayes method was used to predict the dominance effects of SNPs (Zhao et al., 2013; Alves et al., 2019). Additionally, five-fold cross-validation was conducted with 100 iterations for predicting dominance effects and assessing the relationship of heterosis with ∫*RD*. Locally weighted linear regression was performed to determine the relationship between heterosis and genetic distance.

### Partitioning of Genetic Variance Components for Mid-Parent Heterosis

We fitted the extended genomic BLUE model that included the digenic epistatic and dominance effects to estimate the genetic variance components of MPH (Zhou et al., 2012; Jiang et al., 2017; Boeven et al., 2020). The model is shown below:


(5) $$y=g_{d}+g_{a\,a}+g_{a\,d}+g_{d\,d}+e$$

Here, *y* represents the MPH vector of every hybrid, *g*_*d*_, _  g_dd_, *g*_*ad*_, and *g*_*aa*_ indicate genetic values of the vectors related to dominance, dominance-by-dominance, additive-by-dominance, and additive-by-additive effects, and *e* represents the residual. It is assumed that $g_{d}∼N\,\left(0,K_{d}\,\delta_{d}^{2}\right)$, $g_{a\,a}∼N\,\left(0,K_{a\,a}\,\delta_{a\,a}^{2}\right)$, $g_{a\,d}∼N\,\left(0,K_{a\,d}\,\delta \,a_{d}^{2}\right)$, $g_{d\,d}∼N\,\left(0,K_{d\,d}\,\delta_{d\,d}^{2}\right)$, and $e∼N\,\left(0,T\,T^{\prime }\,\delta_{e}^{2}\right)$ in the formula, where *K*_*d*_,*K*_*aa*_, *K*_*ad*_, and *K*_*dd*_ indicate the kinship matrices derived from the markers of different genetic effects, *T* indicates the *lineartransformationr*×(*r* + *s*) matrix from the original trait vectors to the MPH vector, *r* indicates the hybrid number, and *s* represents the parental line number. Next, the *F* metric was used for calculating kinship matrices derived from the markers, resulting in model non-orthogonal parametrization (Jiang et al., 2017). The multi-kernel approach was adopted to estimate the variance components $\delta_{d}^{2}$, $\delta_{a\,a}^{2}$, $\delta_{a\,d}^{2}$, and $\delta_{d\,d}^{2}$ using the R package BGLR (Pérez and De Los Campos, 2014), following previously published settings (Jiang et al., 2017).

### Estimation of Heterotic Effects

For a locus, its heterotic effect represents its genetic contribution to MPH and also the combination of the self-dominance effect and epistatic interaction effect with the entire genetic background (Jiang et al., 2017). Specifically, *Q* was assumed to be the entire QTL set for each phenotypic trait. QTL was assigned a value of 0, 1, or 2, based on the selected allele number at each locus. For each hybrid, *R*_*kl*_(*k*,*l* =  0or 2) was denoted as the locus subset, where the male and female parents had the genotypes *l* and *k*, respectively_. _ For *i*, *j* ∈ *Q* and i ≠ j, *d*_*i*_ is assumed as the dominance effect of the *i*th QTL, while *aa*_*ij*_, *ad*_*ij*_, and *dd*_*ij*_ represent the additive-by-additive, additive-by-dominance, and dominance-by-dominance epistatic effects between *i*th and *j*th QTL. For the *i*th locus, its heterotic effect is shown below:


(6) $$h_{i}=\left\{ \begin{array}{c} d_{i}-\frac{1}{2}\,\sum_{j\in R_{20}}a\,a_{i\,j}+\frac{1}{2}\,\sum_{j\in R_{02}}a\,a_{i\,j}+\frac{1}{2}\,\sum_{j\in R_{22}}a\,d_{i\,j}\\ -\frac{1}{2}\,\sum_{j\in R_{00}}a\,a\,d_{i\,j}+\frac{1}{2}\,\sum_{j\in R_{20}\,\cup R_{02}}d\,d_{i\,j}\,i\,f\,i\in R_{20}\\ d_{i}-\frac{1}{2}\,\sum_{j\in R_{02}}a\,a_{i\,j}+\frac{1}{2}\,\sum_{j\in R_{20}}a\,a_{i\,j}+\frac{1}{2}\,\sum_{j\in R_{22}}a\,d_{i\,j}\\ -\frac{1}{2}\,\sum_{j\in R_{00}}a\,a\,d_{i\,j}+\frac{1}{2}\,\sum_{j\in R_{20}\,\cup R_{02}}d\,d_{i\,j}\,i\,f\,i\in R_{02}\\ \frac{1}{2}\,\sum_{j\in R_{20}\,\cup R_{02}}a\,d_{i\,j}\,i\,f\,i\in R_{22}\\ \frac{1}{2}\,\sum_{j\in R_{20}\,\cup R_{02}}d\,d_{i\,j}\,i\,f\,i\in R_{00} \end{array}\right.$$

According to the definition, the MPH value of the hybrid represents the summation of all heterotic effects among polymorphic loci.

### Genome-Wide Scanning for Significant Heterotic Effects

A three-step process proposed by Jiang et al. (2017) was used to detect significant heterotic effects. First, we conducted genome-wide association mapping to identify significant component effects. Additionally, a standard linear mixed model was constructed along with the kinship matrix derived from the markers to control the structure of various polygenic background effects and relatedness levels (Yu et al., 2005). Assuming the presence of epistasis, a model that controls polygenic background effects, including epistatic and main effects, needs to be constructed (Xu, 2013; Jiang et al., 2017). This model is shown below:


(7) $$y=m\,\alpha +g_{d}+g_{a\,a}+g_{a\,d}+g_{d\,d}+e$$

Here, *y*, *g*_*d*_, *g*_*aa*_, *g*_*ad*_, *g*_*dd*_, and *e* represent the same parameters as those presented in Equation 5. Particularly, α represents the dominance effect of a marker or the epistatic interaction effect of a pair of markers, and *m* represents the relevant coefficient. We assumed α to be an unknown fixed parameter, while the rest were assumed to be the same as those in Equation 5. We converted this model into a standard linear regression model for calculating efficiency, where only the residual terms were random. The converted model was similar to the original one as long as the effect of different parameters on the estimation of the variance components was negligible (Lippert et al., 2011; Xu, 2013). Next, we performed the *F* test to assess the significance of the effect of α (Jiang et al., 2017).

Second, we integrated the significant component effects into the heterotic effects based on Equation 6. We set each non-significant effect as zero.

Third, for all loci, we analyzed their heterotic effect *h*_*i*_ using the permutation test. Specifically, we predicted the hybrid absolute MPH values through their respective heterotic effects. Then, we determined Pearson’s correlation coefficient between the actual and estimated MPH values and conducted permutation tests for the correlation coefficients.

In steps one and three, we examined the genome-wide threshold for the *p*. For the temperate by tropical panel, heterotic and dominance effects were tested by Bonferroni corrected threshold at *p* = *0.001*/*n*. In contrast, the additive-by-additive, additive-by-dominance, and dominance-by-dominance epistatic effects were tested at *p* < *0.001*/*[n(n-1)]*, where *n* denotes the tested number of SNPs (Holm, 1979; Jiang et al., 2017). The small population size restricted the power to detect significant effects for the temperate by temperate panel. Thus, the threshold for the heterotic and dominance effects in GWPP was set at *p* < *0.05/n*, whereas the epistatic effects were tested at *p* <*0.05*/*[n(n-1)]*.

### Omics Network Analysis and Gene-Set Enrichment

To further interpret the genetic structure and identify the important candidate epistatic genes for the two tested traits, the genes adjacent to the significant SNPs less than 55 kb were considered to be the candidate genes, while the length of linkage disequilibrium (LD) decay was 245 kb at *r*^2^ = 0.1 (Jung et al., 2004). In this study, the MaizeGDB database^1^ was used to convert candidate gene names from AGPv3 to AGPv4, and the candidate genes were annotated based on the data from the MaizeGDB database (Harper et al., 2016). A seed indicated the input gene used to query protein–protein interactions (PPI), and the interactome comprised its direct interactor genes. The first layer network comprised the seeds; the second layer network comprised all interactors that interacted with the seeds; the specific network was composed of the first + second layer interactomes. The candidate genes were assigned to PPI using the Maize Interactome Platform (MIP) developed in another study (Han et al., 2020), and the first layer network was constructed based on the interaction of the candidate genes with each other. Each gene in the first layer network acted as seeds, which were used to query the direct interactor genes in the MIP Network Creation tool. Then, all of their genes were selected with the MIP Slim-interactive Omics Network tool to classify them into corresponding modules. The direct interactor genes were used to compose the second layer network. The gene network obtained from MIP was imported into Cytoscape version 3.7.2 for further analysis and displayed. The genes in the different modules were then assigned to perform Gene Ontology under the categories of biological process (GO-BP) to identify biological functions with a false discovery rate (FDR) less than 0.05, based on the online AgriGO Singular Enrichment Analysis tool^2^ (Tian et al., 2017).

## Results

### Broad Breeding Germplasm From Temperate and Tropical Regions to Determine Heterosis

**FIGURE 1:**
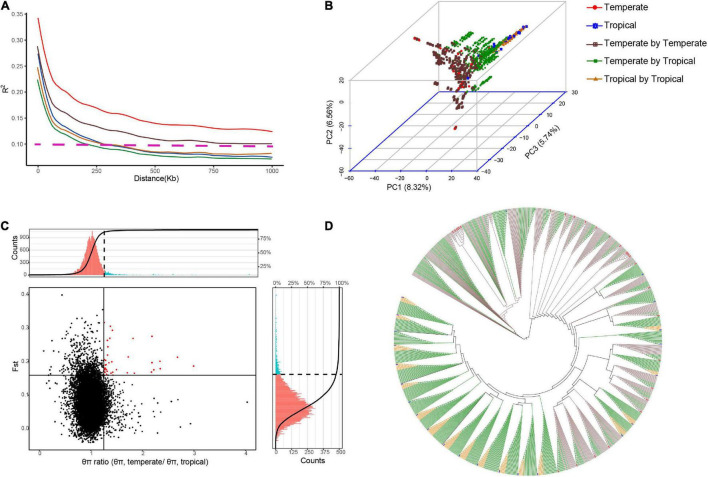
Genetic diversity identified among the hybrids in multiple hybrid populations. Population structure and LD were analyzed for 28 temperate parents, 23 tropical parents, and 1,154 hybrids from the multiple hybrid populations. **(A)** LD decay (*R*^2^) is plotted as a function of the genetic map distance based on pairwise correlations of the LD phase. Horizontal dotted lines represent LD decay lower than 0.1. **(B)** Principal component analysis (PCA) based on the maximum likelihood method. The numbers in the parentheses are genotypic variance proportions explained with the PC1, PC2, and PC3. **(C)** Genome-wide selective sweep analysis on tropical and temperate maize groups. Vertical and horizontal solid lines represent threshold lines for the top 5% of *θπ* ratios and *F*_*ST*_ values, respectively. Red dots in the right upper sector indicate selective signatures for temperate maize lines. The portions of histograms in red and blue colors indicate the *θπ* ratio (upper) and the *F*_*ST*_ (right) values above the thresholds. **(D)** Neighbor-joining tree based on genetic distances.

We produced a multiple-hybrid population of maize with 1,154 hybrids from three subpopulations. The largest genetic distance was identified between the temperate and tropical lines (*p* < 0.001; 0.38), followed by the groups of the tropical (0.36) and temperate lines (0.35). The genetic diversity in the intergroup was also determined, and a faster LD decay was found in the temperate by tropical hybrid set than in the other two hybrid sets. The PCA confirmed the differences among the three hybrid sets, with three final patterns being distributed in different spaces. Using an NJ phylogenetic tree, cluster analysis based on GD estimates identified 49 groups, and the hybrids derived from a parent were mostly clustered into the same group (Figure 1). The *F*_*ST*_ and *θπ* for the entire maize genome were observed from the volcano plot. We discovered 125 candidate selective-sweep areas, which covered 0.61% of the maize genome.

### Higher Level of Heterosis Identified in Temperate by Tropical Hybrids

We evaluated the heterosis of DTS and GWPP for the temperate by temperate and temperate by tropical hybrids from eight agro-ecological sites and the tropical by tropical hybrids in two agro-ecological sites. The heterosis for both GWPP and DTS were normally distributed (Supplementary Figures 2, 3). A moderate-to-high heritability was found, with a value of 0.79 for the temperate by temperate set and a value of 0.84 for the temperate by tropical set for GWPP (Supplementary Figure 4). The broad-sense heritability for GWPP was 0.63 for the temperate by tropical hybrids and 0.77 for the temperate by temperate hybrids. The broad-sense heritability for MPH of DTS was 0.88 for the temperate by temperate hybrids and 0.93 for the temperate by tropical hybrids. The MPH heritability estimates for both traits were higher for the temperate by tropical hybrids (Supplementary Figure 4).

**FIGURE 2:**
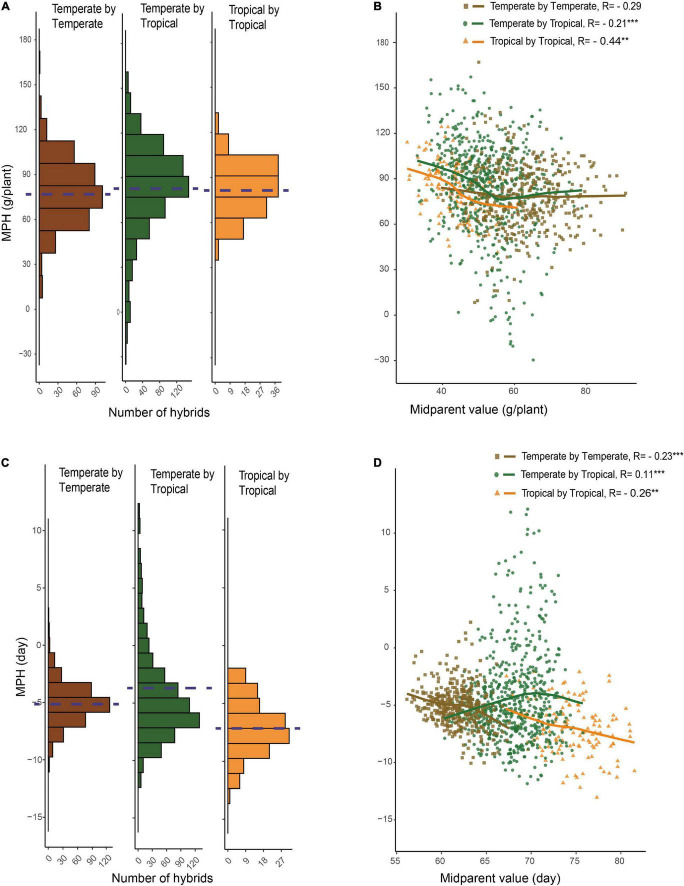
Relationship between hybrid heterosis and mid-parent performance. **(A)** Mid-parent heterosis (MPH) of GWPP for temperate by temperate, temperate by tropical, and tropical by tropical hybrids. **(B)** Relationship between MPH and mid-parent (MP) performance for GWPP. **(C)** Distribution of MPH for days to silking for temperate by temperate, temperate by tropical, and tropical by tropical hybrids. **(D)** Relationship between MPH and MP performance for days to silking. The dashed lines in the histograms indicate the averages.

For GWPP, the mean relative MPH for the temperate by temperate, temperate by tropical, and tropical by tropical hybrids were 128.92, 166.24, and 185.88%, respectively (Supplementary Figure 2; Table 1). The average absolute MPH decreased gradually from the temperate by tropical hybrids to the tropical by tropical hybrids, and finally, to the temperate by temperate hybrids. Hybrids in the temperate by tropical panel had significantly higher absolute MPH than in the temperate by the temperate panel (*p* < 0.01; 84.15 g plant^–1^ vs. 78.48 g plant^–1^; Figures 2A,B). Lower yield in tropical parents might lead to a higher level mid-parent heterosis in the temperate by tropical hybrids, considering that the temperate parents had 46.49% higher GWPP than tropical parents. However, the temperate by tropical hybrids had 4.7% higher absolute BPH than the temperate by temperate hybrids. The average absolute MPH for DTS decreased significantly from the temperate by tropical hybrids (−4.46 days) to the temperate by temperate hybrids (−5.07 days), and finally, to the tropical by tropical hybrids (−7.00 days) (Figures 2C,D). The extent of heterosis showed high specificity for every hybrid set, which could be interpreted either by different interparental genetic distances or the genetic mechanisms for the heterosis between hybrid panels.

**TABLE 1:** Summary statistics for tested traits (GWPP and DTS) and their heterosis.

Source	DTS (days)	GWPP (g plant^–1^)
Temperate line (Min; Max)	61.75 (56.03; 68.61)	62.45 (36.91; 94.32)
Tropical line (Min; Max)	68.23 (59.76; 77.30)	42.63 (29.07; 63.08)
**Temperate by temperate hybrids (*n* = 377)**		
Average (Min; Max)	56.67 (51.67; 65.48)	140.99 (57.26; 217.01)
Average MPH (Min; Max)	−5.07 (−10.06; 2.23)	78.48 (8.36; 166.97)
Average MPH% (Min; Max)	−8.20 (−15.89; 3.53)	128.92 (16.5; 333.64)
Average BPH (Min; Max)	−6.90 (−15.88; 1.83)	70.15 (2.83; 161.59)
Average BPH% (Min; Max)	−10.79 (2.87; −23.15)	103.09 (5.20; 291.53)
**Temperate by tropical hybrids (*n* = 641)**		
Average (Min; Max)	63.48 (52.48; 81.7)	136.73 (35.47; 205.06)
Average MPH (Min; Max)	−4.46 (−11.84; 12.09)	84.15 (−29.69; 157.24)
Average MPH% (Min; Max)	−6.61 (−17.02; 17.46)	166.24 (−45.56; 422.31)
Average BPH (Min; Max)	−10.68 (−22.02; 6.95)	73.45 (−46.86; 149.99)
Average BPH% (Min; Max)	−14.28 (−27.81; 9.58)	125.21 (−56.92; 355.62)
**Tropical by tropical hybrids (*n* = 136)**		
Average (Min; Max)	67.56 (60.77; 76.27)	126.93 (87.26; 165.67)
Average MPH (Min; Max)	−7.00 (−13.05; −2.09)	79.71 (28.7; 124.25)
Average MPH% (Min; Max)	−9.37 (−16.87; −2.78)	185.88 (38.62; 377.9)
Average BPH (Min; Max)	−9.85 (−17.35; −2.88)	70.70 (−15.59; 123.08)
Average BPH% (Min; Max)	−12.67 (−21.65; −3.64)	152.75 (−13.15; 375.15)

*DTS, days to silking; GWPP, grain weight per plant; BPH%, better parent heterosis percentage; BPH, absolute better-parent heterosis; MPH%, mid-parent heterosis percentage; MPH, absolute mid-parent heterosis.*

### Linear Relationship Between Heterosis and Heterotic Genetic Distance

**FIGURE 3:**
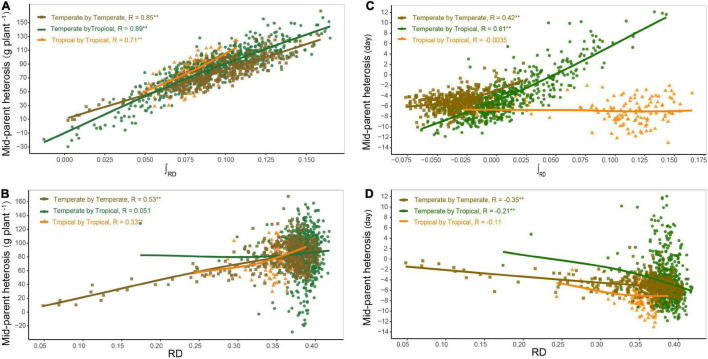
Relationship between genetic distance and heterosis. Panels **(A,B)** show the relationship of mid-parent heterosis (MPH) with heterotic genetic distance (∫*RD*) and Rogers’ distance (RD) for grain yield per plant (GWPP), respectively. Panels **(C,D)** show the relationship of MPH with ∫*RD* and RD for days to silking (DTS), respectively. The regression lines in different colors represent the locally weighted regressions for temperate by temperate (golden), temperate by tropical (green), and tropical by tropical (orange) hybrid sets.

**FIGURE 4:**
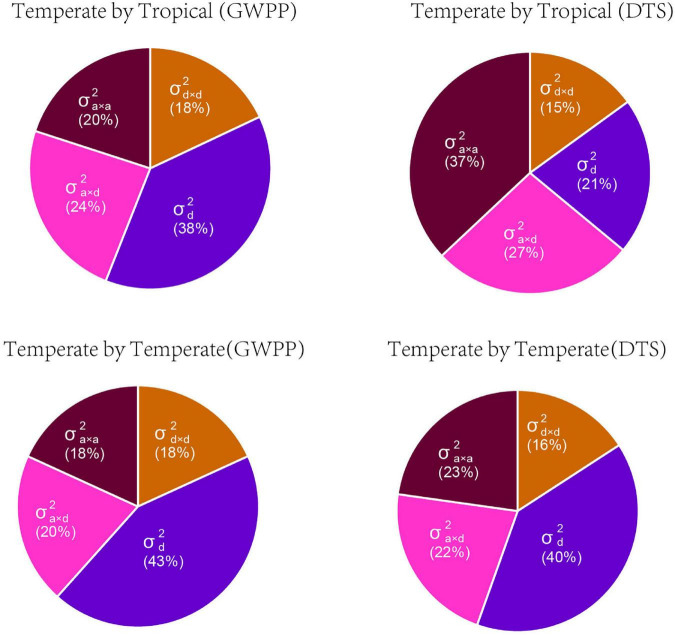
The relative contributions of genetic variance components for mid-parent heterosis for grain yield per plant (GWPP) and days to silking (DTS). Variance components predicted by Bayesian generalized linear regression: $\sigma_{d}^{2}$, dominance variance; $\sigma_{d\times d}^{2}$, dominance-by-dominance variance; $\sigma_{a\times d}^{2}$, additive-by-dominance variance; and $\sigma_{a\times a}^{2}$, additive-by-additive variance.

Based on the dominance model, heterosis for GWPP showed a monotonic increase as the interparental heterotic genetic distance increased, explaining 70.78% of the variation (Figure 3A; Supplementary Figures 5, 6). The unknown part of the variation probably resulted from epistatic effects or the noise. The former was obtained from heterosis variance partitioning with various components, and the results should be interpreted with caution (Huang and Mackay, 2016). For GWPP and DTS, epistasis accounted for 62 and 79% of the total genetic variation in the temperate by tropical hybrids and 57 and 60% in the temperate by temperate hybrids, respectively (Figure 4). The contribution of the dominant effect to the total genetic variance of heterosis was relatively low, and the close relationship between heterotic genetic distance and heterosis was not contradictory. On the contrary, the estimated dominant effect also captured different types of epistatic interactions, as shown by the correlation between the marker-derived dominant kinship matrix and the three types of digenic epistatic effects (Supplementary Tables 2, 3). The correlation between genetic distances and heterosis was weak when the dominant effects were ignored while estimating the genetic distances (Figures 3B,D; Supplementary Figures 5, 6).

Heterosis continuously increased with heterotic genetic distance, and the mean heterosis increased considerably in the temperate by tropical hybrids than in the temperate by temperate hybrids. The regression line of heterosis with heterotic genetic distance for the temperate by tropical hybrids was lower than that for the temperate by temperate hybrids at the beginning, and then the regression line of the temperate by tropical hybrids exceeded that of the temperate by temperate hybrids, increasing almost in parallel with the increase in the heterotic genetic distance (Figure 3A). However, for a given heterotic genetic distance, heterosis performance for GWPP was lower in the temperate by temperate hybrids compared to the temperate by tropical hybrids, but for DTS, it showed an opposite trend (Figure 3C), which might be due to the violation of the assumption that genetic effects should be similar among different hybrid sets when heterotic genetic distances are estimated.

### Epistatic Effects Make Greater Contributions to Heterosis in the Temperate by Tropical Hybrids

Based on the framework proposed by Jiang et al. (2017), we conducted whole-genome prediction of heterosis for GWPP using 644 temperate by tropical hybrids and 377 temperate by temperate hybrids by modeling digenic epistatic and dominance effects. As revealed by five-fold cross-validation with 100 runs, the model predicted 68.43 and 78.56% of heterosis-related genetic variances for GWPP, and 65.93 and 80.76% for DTS, in the temperate by tropical and the temperate by temperate hybrid sets, respectively (Supplementary Table 4). The additive-by-additive model performed better than the dominance effects, with an increase in the genome-wide prediction accuracy by 6.5 and 4.5% in the temperate by tropical hybrids for GWPP and DTS, respectively. However, the combination of the effects in the two models of digenic epistatic and dominance did not affect prediction accuracy, which was probably caused by the high correlation between epistasis and dominance kinship matrices derived from the markers (Supplementary Table 2 and Supplementary Figure 7). Additionally, whole-genome prediction accuracies for GWPP based on the dominance effects outperformed the additive-by-additive effects by 1.46% in the temperate by temperate hybrids, and combining both types of effects did not improve the prediction accuracy, mainly because the dominance model explained the major portion of genetic variance (Figure 4; Supplementary Figure 7). Partitioning the total genetic variance of heterosis into its components described a particularly important role in epistasis (Figure 4), and the additive-by-dominance and additive-by-additive epistasis largely contributed to heterosis in the temperate by tropical hybrids.

**FIGURE 5:**
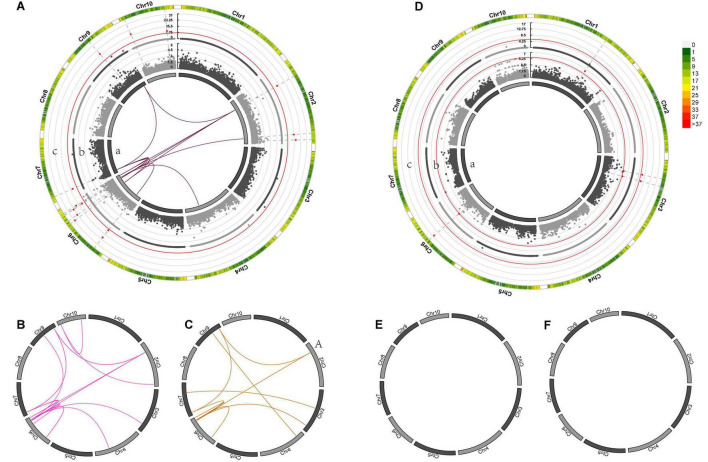
Genetic effects of mid-parent heterosis for maize GWPP in temperate by tropical **(A–C)** and temperate by temperate **(D–F)** hybrids. **(A–F)** In the inner circle, 10 chromosomes are indicated by bars. Gray lines indicate the genetic map locations of SNPs. Color links within the circle indicate significant digenic epistatic interactions, including additive-by-additive [**(A)**a, **(D)**a], additive-by-dominance **(B,E)**, and dominance-by-dominance **(C,F)** interactions. Manhattan plots for the heterotic effects [**(A)**c, **(D)**c] and the dominance effects [**(A)**b, **(D)**b] from genome wide association mapping are presented using Manhattan plots from GWAS. Red lines indicate the threshold of significance. Bar colors denote marker density, with one bar representing a window size of 1 Mb.

Genome-wide association mapping for GWPP discovered four SNP loci with significant dominance effects in the temperate by temperate hybrids and 33 pairs of markers with significant epistatic effects (Figures 5A–F and Supplementary Data Sheets 2, 3) in the temperate by tropical hybrids, including 11 additive-by-additive, 13 additive-by-dominance, and nine dominance-by-dominance interactions (Figures 5A,C). The absence of significant dominance effects in the temperate by tropical hybrids could be partly interpreted by the low contribution (38%) of the dominance effects to the grain-yield heterosis-related genetic variance. The dominance effects in the temperate by temperate hybrids were positive, while 31 of the 33 (93.9%) epistatic effects in the temperate by tropical hybrids were negative. Whether there were significant heterotic QTL effects was determined by the association of the detected heterosis with the estimated contribution of single heterosis QTL, with 14 and 3 heterotic QTL identified in the temperate by tropical and the temperate by temperate hybrid sets, explaining 32.67 and 31.16% of the mean phenotypic variance, respectively (Figures 5Ac,Dc).

**FIGURE 6:**
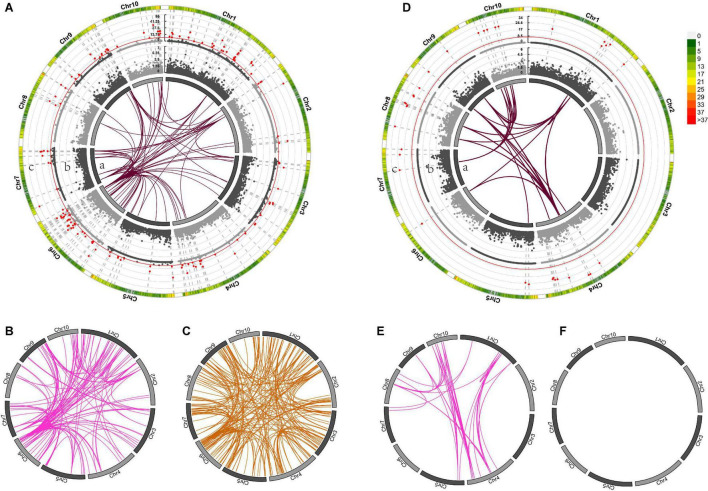
The different mid-parent heterosis genetic effects for days to silking (DTS) in temperate by tropical **(A–C)** and temperate by temperate **(D–F)** hybrids. **(A–F)** In the inner circle, 10 chromosomes are expressed in the form of bars. Gray connector lines indicate the genetic map locations of the SNPs. The colored links within the circle indicate obvious digenic epistatic effects, including additive-by-additive effects [**(A)**a, **(D)**a], additive-by-dominance effects **(B,E)**, as well as dominance-by-dominance effects **(C,F)**. Manhattan plots for the heterotic effects [**(A)**c, **(D)**c] and the dominance effects [**(A)**b, **(D)**b] from genome wide association mapping. Red lines indicate the threshold of significance. The different bar colors denote marker density, with one bar representing a window size of 1 Mb.

Genome-wide association mapping for DTS detected 81 pairs of markers with significant epistatic effects in the temperate by temperate hybrids (Supplementary Data Sheets 4, 5), including 34 additive-by-additive and 46 additive-by-dominance interactions (Figures 6Da,E,F), and 420 marker pairs with significant epistatic effects in the temperate by tropical hybrids, including 93 additive-by-additive, 145 additive-by-dominance, and 182 dominance-by-dominance interactions (Figures 6A–C). The absence of significant dominance effects could partly be interpreted by the low contribution of the dominance effects (21 and 40%) to the DTS heterosis-related genetic variance. Of the 420 epistatic effects identified in the temperate by tropical hybrids, 68 (16.43%) were negative (Supplementary Data Sheet 4), while 33 of the 238 (13.87%) epistatic effects in the temperate by temperate hybrids were negative. A total of 139 and 37 heterotic QTL were identified in the temperate by tropical and the temperate by temperate hybrid sets (Figure 8A; Supplementary Data Sheet 4), respectively, which explained 31.9 and 50.71% of the phenotypic variance on average (Figures 6Ac,Dc).

### Candidate Genes and Epistasis Identified in the Temperate by Tropical Hybrids

**FIGURE 7:**
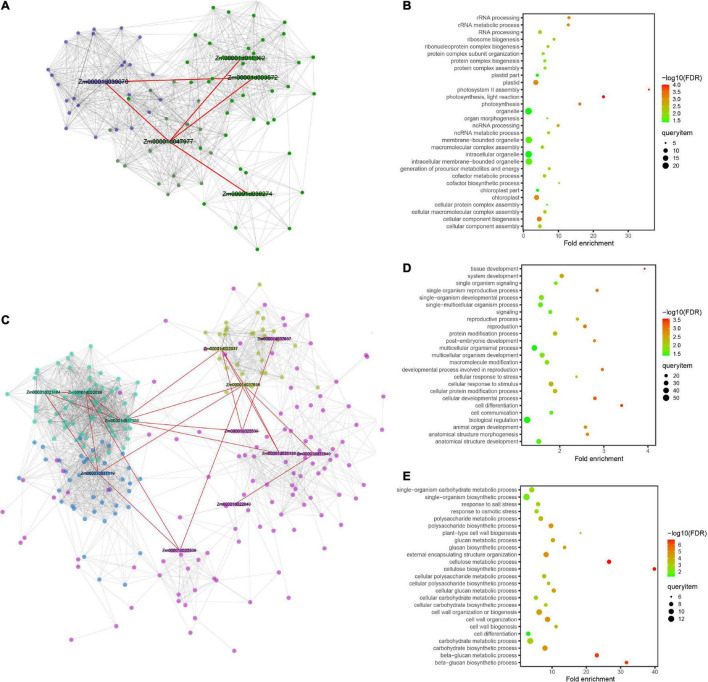
Protein–protein interaction network and enrichment information of the modules for grain weight per plant (GWPP) and days to silking (DTS) in maize. Results are shown for the GWPP **(A,B)** and DTS **(C–E)**. **(A)** The *Zm00001d047977* gene interaction network has two modules and each module is marked with different colors. The red line indicates the protein–protein interaction network for the significant gene located by genome-wide association analysis. **(B)** The dark blue module shows enrichment in multiple developmental processes for GWPP. **(C)** The gene interaction network, including *Zm00001d037637*, *Zm00001d037640*, and *Zm00001d037636*, has four modules, marked with different colors, three of which have genes enriched in multiple developmental processes. **(D,E)** The pink and turquoise modules show enrichment in multiple developmental processes for DTS. The hexagon represents the core epistatic genes (first layer nodes), and the red line indicates the relationships of the core epistatic genes. Different colors represent module analysis using the Maize Interactome platform (second layer nodes).

The interactomes associated with digenic epistasis showed that gene interactions were involved in various biological processes. A total of nine million interactions spanning all levels of genetic information flow across the entire maize lifecycle have been elucidated using the Maize Interactome platform (Han et al., 2020). For GWPP in the temperate by tropical hybrids, a significant locus (Chr2.47114632) was analyzed, along with the other five epistatic loci. A total of 16 candidate genes within 55 kb widows of the epistatic loci were identified using the online Maize Interactome platform PPI Network tool, and five core genes were found with interactions (Figure 7A). The five genes were used to build a protein network using the Network Creation instrument, with 90 interacted genes identified. The 90 genes were then analyzed using the online Maize Interactome platform Slim-interactive Omics Network tool, which could be classified into two modules. The first module included 25 genes enriched with GO-BP terms, which could be classified into three functional categories, photosynthesis system (photosystem II assembly, photosynthesis, light reaction, and photosynthesis), positive regulation of transcription (nucleic acid templated transcription modulation, transcription modulation, and RNA biosynthetic process modulation), and protein synthesis (protein complex assembly, protein complex biosynthesis, ribonucleoprotein complex biogenesis, protein complex subunit organization, and ribosome biogenesis) (Figure 7B).

**FIGURE 8:**
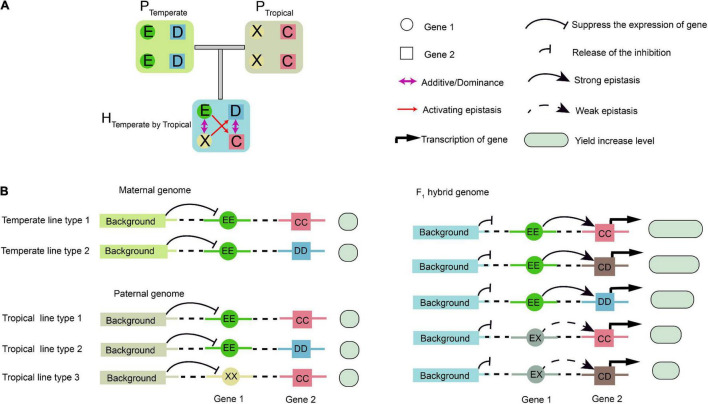
Schematic illustration of epistatic effects. **(A)** Epistasis does not occur between temperate parental (P_Temperate_) or tropical parental (P_Tropical_) lines due to their homozygous genotypes. When two types of inbred lines are crossed, the genetic background in the hybrid changes, resulting in epistatic interaction between the two genetic loci, Gene 1 and Gene 2, which have additive and dominant effects. **(B)** A putative model for interpretation of the interactions between Gene 1, Gene 2, and background genes. In the parental inbreds, background genes repress the transcription of Gene 1. In the hybrid, the repression effect on Gene 1 is relieved, and its transcripts activate the expression of Gene 2. Then, transcription of Gene 2 is activated with different expression levels. The genetic background determines upstream protein–protein interactions specific to the F_1_ hybrids, and the E, D, X, and C in a circle or square represent different alleles. The yield associated with the epistasis is measured by the sizes of the empty circles and boxes in light green.

For DTS in the temperate by tropical hybrids, a significant locus (Chr6.47114632) with the other nine epistatic interaction loci were analyzed. A total of 40 candidate genes within 55 kb widows of their epistatic loci were analyzed; 12 core genes showed interactions (Figure 7C). The 12 core genes were used to build a protein network, with 296 interacted genes and four modules identified. The first module with 64 genes was enriched with GO-BP terms, which were involved in various growth and development processes, such as “cell differentiation,” “single-organism development process,” “multicellular organism development,” “cell development process,” “cell response to stress,” “reproductive process,” and “cell response to stimulus” (Figure 7D). The third module with 31 genes was enriched with GO-BP terms, which supported various biosynthesis processes, including the “cellular carbohydrate biosynthesis process,” “glucan metabolic process,” and “beta-glucan biosynthesis process,” and the abiotic stress responses, including the “salt stress response” and “osmotic stress response” (Figure 7E).

### Epistasis Activation Contributes to Greater Heterosis in the Temperate by Tropical Hybrids

In the parental population, the homozygous background was assumed to suppress the expression of Gene 1 (Figure 8A). In the F_1_ hybrids, however, such suppression was relieved due to the complementation of heterozygosity, which resulted in the activation of epistasis between Gene 1 and Gene 2. Thus, Gene 1 and Gene 2 might be considered to be epistatically controlled QTL (Figure 8A). Using the half-sib hybrids generated between the temperate and tropical maize, the genetic mechanisms of heterosis for single-crosses might be explained. The alleles at Gene 1 had additive or dominant effects, but they were undetectable by GWAS in the temperate population due to the absence of the weak activating allele and no phenotypic difference. However, a phenotypic difference was found in the temperate by tropical hybrids, as shown by GWPP. One possible mechanism for the difference identified using the two sets of hybrids might involve transcriptional regulation and PPI, that is, the upstream PPI network in the hybrids released the inhibition of background Gene 1, and the product of Gene 1 further activated the expression of Gene 2 (Figure 8B).

**FIGURE 9:**
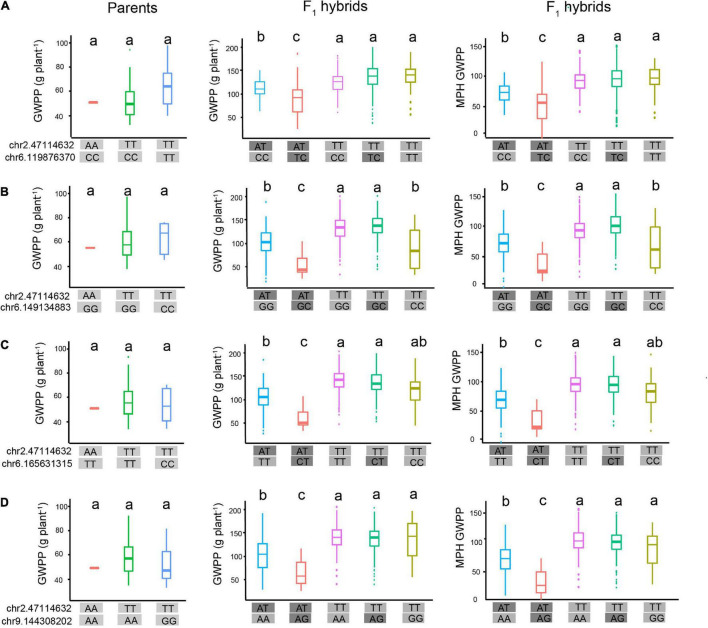
Hypothetical model of epistatic effects affecting heterosis of grain weight per plant (GWPP). Epistatic interaction between chr2.47114632 and four loci contributes to the heterotic performance of GWPP in F_1_ hybrids. **(A)** In the parental lines (left panel), the three haplotypes showed similar GWPP, indicating that there was no interaction between chr2.47114632 and chr6.119876370. In the F_1_ hybrids (middle panel), the genotype chr2.47114632-TT (rose red boxes, green boxes, fluorescent green boxes) exhibited higher GWPP, indicating that chr2.47114632-TT activated a strong positive additive/dominance induced epistatic effect with the locus chr6.119876370 in F_1_ hybrids, and chr2.47114632-TA exhibited lower GWPP (blue boxes, red boxes), indicating that chr2.47114632-TA activated a weak epistatic effect with the locus chr6.165631315 in F_1_ hybrids. Panels **(B–D)** present the epistatic interactions between chr2.47114632 and three other loci (chr6.149134883, chr6.165631315, and chr9.144308202, respectively), contributing to the heterotic performance of GWPP in F_1_ hybrids. The a, b, and c above the haplotype stick bars represent multiple comparisons by the Student–Newman–Keuls test with α = 0.05.

The molecular interpretation of the above-mentioned epistasis can be exemplified by the QTL chr2.47114632 and the epistatic QTL chr6.119876370. We hypothesized that this epistatic interaction agreed with the scenario illustrated in Figure 8B, because the genotype chr2.47114632-AA was detected in only three tropical parents. Genetic analysis of the two loci, chr2.47114632 and chr6.119876370, indicated that the homozygous genotype at chr2.47114632 was probably inhibited by the background genotype in the parent lines. In the F_1_ hybrids, however, a specific of PPI occurred when two parental genomes combined, as the proteome of each parent might provide novel interacting partners (Li et al., 2020). As a result, the repression at chr2.47114632 was removed, which allowed the expression of the chr2.47114632 allele. The full expression of the genotype chr2.47114632-TT activated the chr6.119876370 allele to fully express GWPP, and the genotype chr2.47114632-AT activated the chr6.119876370 allele to weakly express GWPP (Figure 9A). The three tropical lines carrying the AA genotypes at chr2.47114632 exhibited lower GWPP in the parent lines, and in the F_1_ hybrids, the genotype chr2.47114632-AT also had a relatively low GWPP, indicating that the genotype chr2.47114632-AA was unfavorable to GWPP. Similar interaction occurred between chr2.47114632 and three other loci (Figures 9B–D), indicating that chr2.47114632 might host an important regulatory gene involved in multiple biological processes in the formation of GWPP.

## Discussion

The hybrids generated from inbred lines in the earlier generation showed higher levels of fitness and heterosis (Rhode and Cruzan, 2005). Various models have been proposed to explain heterosis, including dominance, overdominance, and epistasis with complex allelic, intragenomic, and intergenomic interactions (Birchler et al., 2010; Kaeppler, 2012). According to recent studies on rice and maize (Huang et al., 2015; Yang et al., 2017), most heterotic genes exhibit incomplete or partial dominance. However, the relationship between multiple-locus epistasis and heterosis has been under-studied due to the complexity and difficulty in profiling a bonafide and comprehensive interactome in F_1_ hybrids (Li et al., 2020). Additionally, the overall contribution of individual small-scale mutations to heterosis was mostly weaker than the effects arising from genetic variations in major alleles, but the cumulative effects from dozens to hundreds of small-scale mutations might contribute to the undiscovered component of heterosis. The unique design of multi-hybrid populations derived from the temperate and tropical lines provides a chance to understand the genetic mechanism of heterosis. Furthermore, tropical maize with favorable alleles for abiotic and biotic stress resistance should be used to increase genetic diversity and accumulate favorable QTL with minor effects. With such a design, the alleles in the tropical lines with minor positive or negative effects on fitness might have been retained, resulting in the accumulation of many minor favorable or deleterious mutations. Therefore, the founder lines used in our study can be used to detect such rare mutations to increase the understanding of the contribution of minor gene interactions to heterosis.

Hybrid breeding largely depends on heterosis, which can be determined by the genetic distance between parental lines (Tian et al., 2016). The level of heterosis is generally related to parental genetic diversity (Tian et al., 2019). However, some studies have found a weak or no relationship between marker diversity and yield (Oyekunle et al., 2015; Boeven et al., 2020). Therefore, genetic diversity is important but is not enough to give rise to desirable heterosis performance (Fu et al., 2014). Heterosis in the temperate by tropical and temperate by temperate hybrid sets increased with the heterotic genetic distance, and the temperate by tropical hybrids showed a relatively higher level of heterosis for grain yield. This indicated that intensive selection by breeding, possible genetic drift, or both had created a divergence between the two maize groups, resulting in an increase in heterosis associated with a non-dominant effect. This is supported by the fact that GWPP showed a weak correlation with plant height (*r* = 0.07), grain number per row (0.25), ASI (*r* = 0.17), and hundred-grain weight (0.34) for tropical parents, due to the negative correlation between mid-parent performance and heterosis (Supplementary Figure 8). Genetic divergence occurs in isolated populations, which decreases the hybrid fitness, but there are many instances where hybrids show a higher degree of fitness than their parents (Dagilis et al., 2019). Thus, in this study, we adopted the quantitative genetic framework proposed by Jiang et al. (2017) to understand possible genetic causes for heterosis and identified epistatic effects that contributed to stronger heterosis in the temperate by tropical hybrids. Additionally, our results indicated that the most prevalent heterosis was controlled by epistatic genes in the tropical by temperate hybrids, and the prevalence of multiple-locus epistatic interactions might explain the genetic control of hybrid vigor in general. This is analogous to Fisher’s geometric model where heterosis is involved in the crosses between distantly related inbred lines, and the fitness values of the hybrids include epistatic effects among many loci (Simon et al., 2018; Dagilis et al., 2019).

The complexity of the digenic epistatic network identified in this study suggests that many rare genes with minor effects were modulated by the core genes for yield heterosis. The signals of selection identified between the temperate and tropical hybrids indicated the presence of polygenic heterogeneity along the whole genome (Figure 1C), and in plants, GWPP and DTS are some examples of traits with a polygenic basis. Combining GWAS hits with the PPI networks, we found that the epistatic genes identified by GWAS interacted with many undetected background genes, indicating that genomic variations might cause the expression of many minor differential genes and molecular interactions between the two parents. Additionally, the PPI genes for GWPP were mainly related to photosynthesis, regulation of transcription, and protein complex assembly, suggesting that enhanced photosynthetic or biological pathways during development might be associated with hybrid vigor. The maize yield heterosis results from multiple QTL effects accumulated during the development of the hybrid plants (Xiao et al., 2021). In our study, many epistatic QTL were discovered simultaneously within network pathways for different traits, with nine heterosis QTL identified in common for GWPP and DTS in the temperate by tropical hybrids (Supplementary Figure 9). The floral transition might be a key stage in the formation of heterosis, where epistatic QTL are activated by parental contributions of alleles that counteract the recessive deleterious maternal alleles (Xiao et al., 2021). The correlation of heterosis between yield and many other traits suggests that yield heterosis reflects both the cumulative influence of heterosis with minor effects for many traits and the interaction through various molecular mechanisms (Flint-Garcia et al., 2009). Although our data and results were compelling, our study had some limitations. We obtained GWAS hits from the experimental sets of different sample sizes, which could have introduced bias. However, we mitigated this effect by taking the different significance thresholds for each experimental set. We suggest that future studies should use other methods to validate our candidate core genes, by deep sequencing to identify rare variants and wet-lab experiments to validate yield relevance.

Our GWAS and haplotype analyses indicate that epistasis contributes to the greater heterosis identified in the tropical by temperate hybrids. Epistasis might be related to various molecular interactions, and single or combined alterations of hybrid biological networks might contribute to heterosis to different degrees (Li et al., 2020). The complementary dominant gene expression of hybrids between transcriptional regulatory networks involved in biological pathways across developmental stages contributes to heterosis (Liu et al., 2021; Li et al., 2022). Many minor complementary dominant genes activate epistatic interactions with their PPI genes, resulting in a cascade of amplified phenotypic effects in the hybrids. Multiple alleles at Chr2.47114632 had different types of epistatic effects, producing a series of amplified effects on upstream and downstream gene regulation. This might explain why we detected many epistatic QTL through GWAS with a very strict significance threshold, although the overall contribution of complementary harmful alleles to heterosis was also found. The gene GRMZM2G147158 encoding calmodulin-binding protein 60 C (CBP60C) located at chr2.47114632 belongs to a plant-specific protein family that plays an important role in plant growth/development and biotic/abiotic stress responses, and CBP60 is a central transcriptional activator of immunity in *Arabidopsis* and positively regulates salicylic acid and abscisic acid biosynthesis (Wan et al., 2012; Li et al., 2021; Yu et al., 2021). Our results suggest that the concurrent detection of chr2.47114632 with the other four loci arose from different epistatic effects, and thus, the epistatic genes might be a central transcriptional activator to regulate downstream gene expression.

Designing optimal genotypic combinations and increasing the favorable alleles among parental lines might further enhance hybrid performance. In this study, we found that increasing parental heterotic genetic distance is necessary for maximizing heterosis. The mid-parent values only contributed to a fraction of hybrid performance in maize, while the lines with high GCA for yield were more suitable as parents for hybrid breeding (Supplementary Figure 10). Conversely, the mid-parent values accounted for a large proportion of hybrid performance in wheat hybrids (Zhao et al., 2013; Jiang et al., 2017; Boeven et al., 2020). In this study, mid-parent values were relatively stable across all cross combinations, and the increase in the performance of the hybrids was mainly contributed by mid-parent heterosis (Supplementary Figures 10A,B). With the increase in the mid-parent values, heterosis decreased at the beginning and then remained stable. Thus, whether hybrid yield can be improved with a further increase in the parental genetic distance while maintaining a certain level of parental performance is uncertain. With known heterotic groups, maize breeders usually use pedigree breeding for breeding inbred lines, by which dominant genes between parental lines from two heterotic groups can complement each other. However, early-generation selection lines are characterized by a high level of heterozygosity, making it impossible to select such dominant loci because of lower favorable allele frequencies and the complexity of epistasis. Thus, in the early generations, abiotic and biotic stress resistance, plant maturity, plant height, and other traits are prioritized for selection. In later generations, a large effective population is crucial for selecting desirable combinations of genotypes for grain yield.

## Data Availability Statement

The original contributions presented in this study are included in the article/Supplementary Material, further inquiries can be directed to the corresponding authors.

## Author Contributions

YX and ZS conceived and designed the experiments and wrote the manuscript. ZS, HW, and ZZ performed the experiments. ZS, YY, XL, and ZL analyzed the data. All authors contributed to the article and approved the submitted version.

## Conflict of Interest

The authors declare that the research was conducted in the absence of any commercial or financial relationships that could be construed as a potential conflict of interest. The reviewer KB declared a shared parent affiliation with the several authors ZS, HW, YY, XL, ZL, and YX to the handling editor at the time of review.

## Publisher’s Note

All claims expressed in this article are solely those of the authors and do not necessarily represent those of their affiliated organizations, or those of the publisher, the editors and the reviewers. Any product that may be evaluated in this article, or claim that may be made by its manufacturer, is not guaranteed or endorsed by the publisher.

## Footnotes

1
http://www.maizegdb.org

2
http://systemsbiology.cau.edu.cn/agriGOv2
