# Supplementary material for: Epistasis Activation Contributes Substantially to Heterosis in Temperate by Tropical Maize Hybrids
Source: Front Plant Sci. 2022 Jul 11;13:921608. doi: 10.3389/fpls.2022.921608 (PMC9313604; doi:10.3389/fpls.2022.921608)
Supplement: Supplementary file 1 [file Data_Sheet_1.docx]

***Supplementary Material***


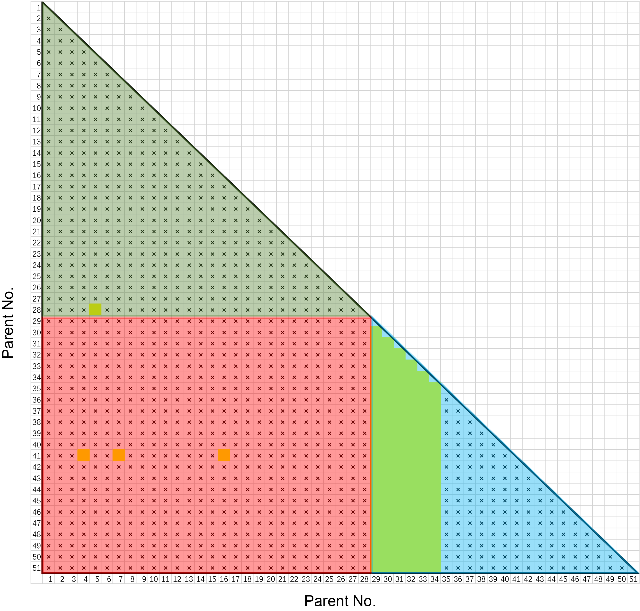


**Figure. S1 Schematic illustration of the multiple-hybrid population with diallel and NC Ⅱ mating design.** (**A**) Temperate by temperate, diallel mating design with 28 temperate lines resulting in 377 hybrids were used for analyses. These temperate by temperate hybrids were evaluated at three locations. (B) Temperate by tropical, incomplete factorial mating design with 28 temperate and 23 tropical lines, resulting in 641 hybrids were used for analyses at the three locations as the temperate by temperate hybrids. (C) Tropical by tropical, diallel mating design with 16 tropical lines resulting in 96 hybrids that were tested at Sanya and Jinghong. The yellow part indicates that the material is missing.


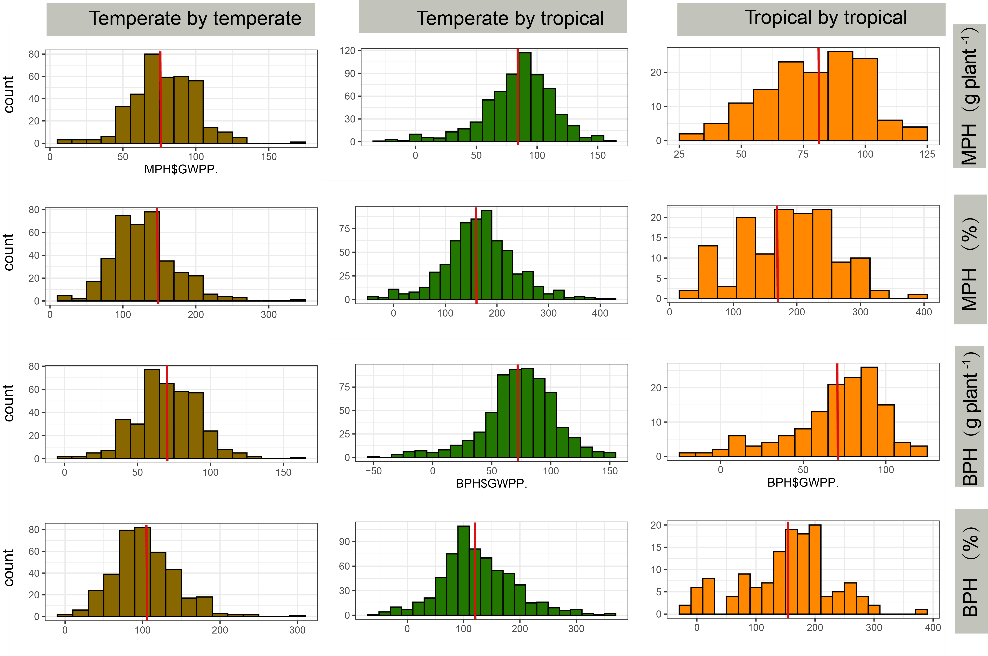


**Figure. S2 Distribution of heterosis for grain weight per plant.** Distribution of absolute and relative mid-parent heterosis (MPH) and absolute and relative better-parent heterosis (BPH) for grain weight per plant for the temperate by temperate, temperate by tropical, and tropical by tropical sets of hybrids. The red vertical line indicates the mean.


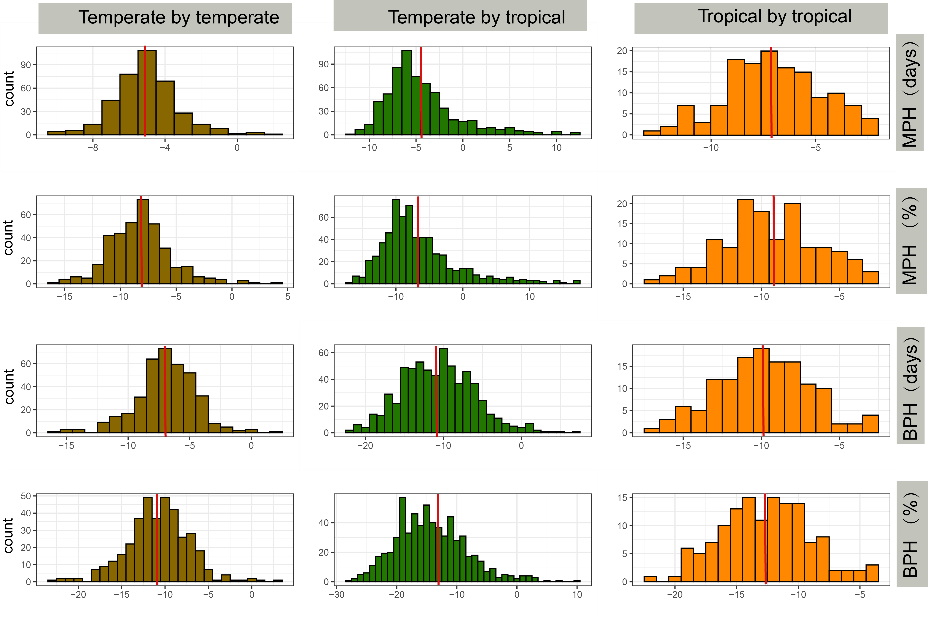


**Figure. S3 Distribution of heterosis for days to silking.** Distribution of absolute and relative mid-parent heterosis (MPH) and absolute and relative better-parent heterosis (BPH) for days to silking for the temperate by temperate, temperate by tropical and tropical by tropical sets of hybrids. The red vertical line indicates the mean.


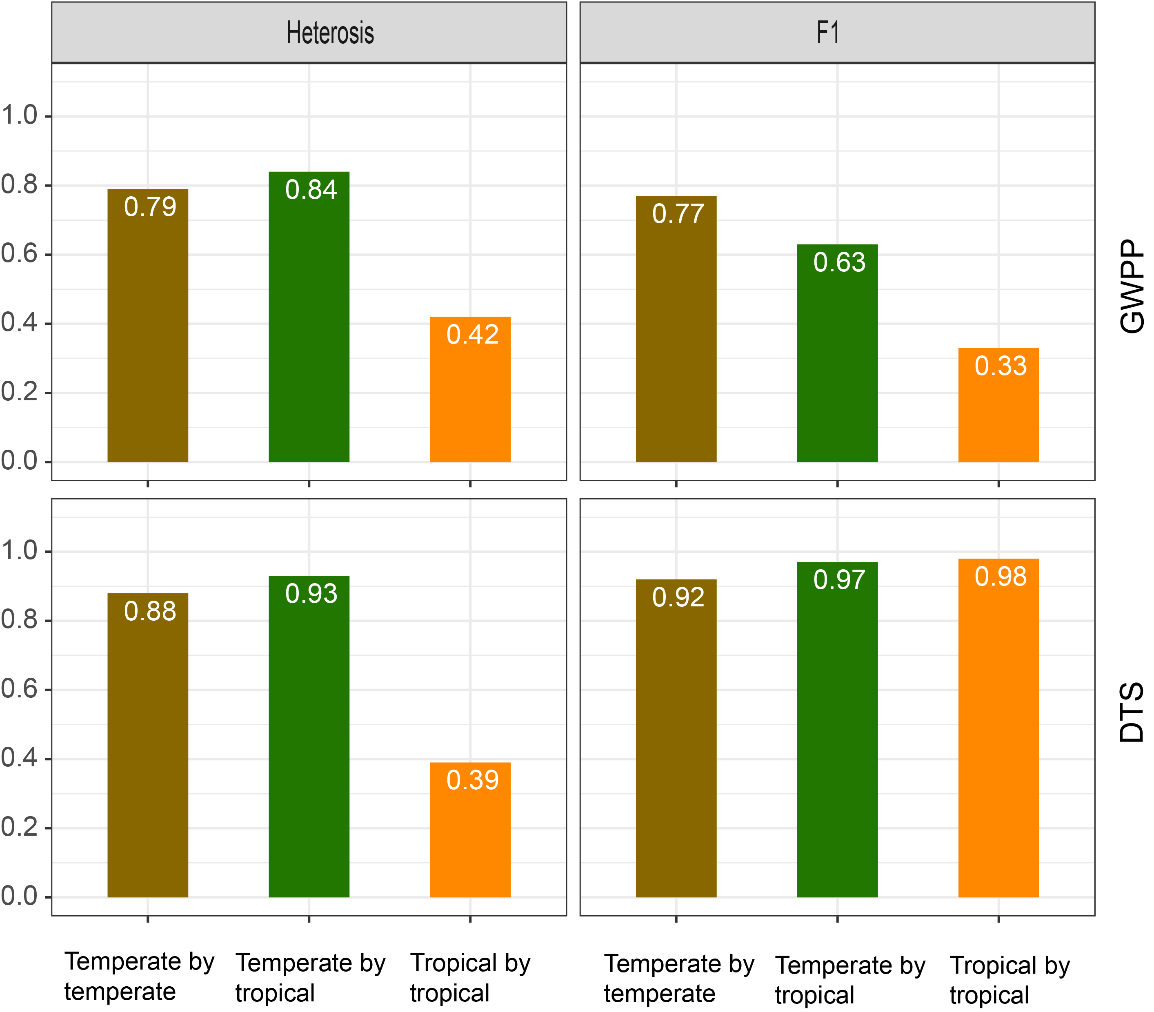


**Figure. S4 Heritability estimates of grain weight per plant (GWPP) and days to silking (DTS) for the populations of hybrids (F_1_) as well as for mid-parent heterosis.**


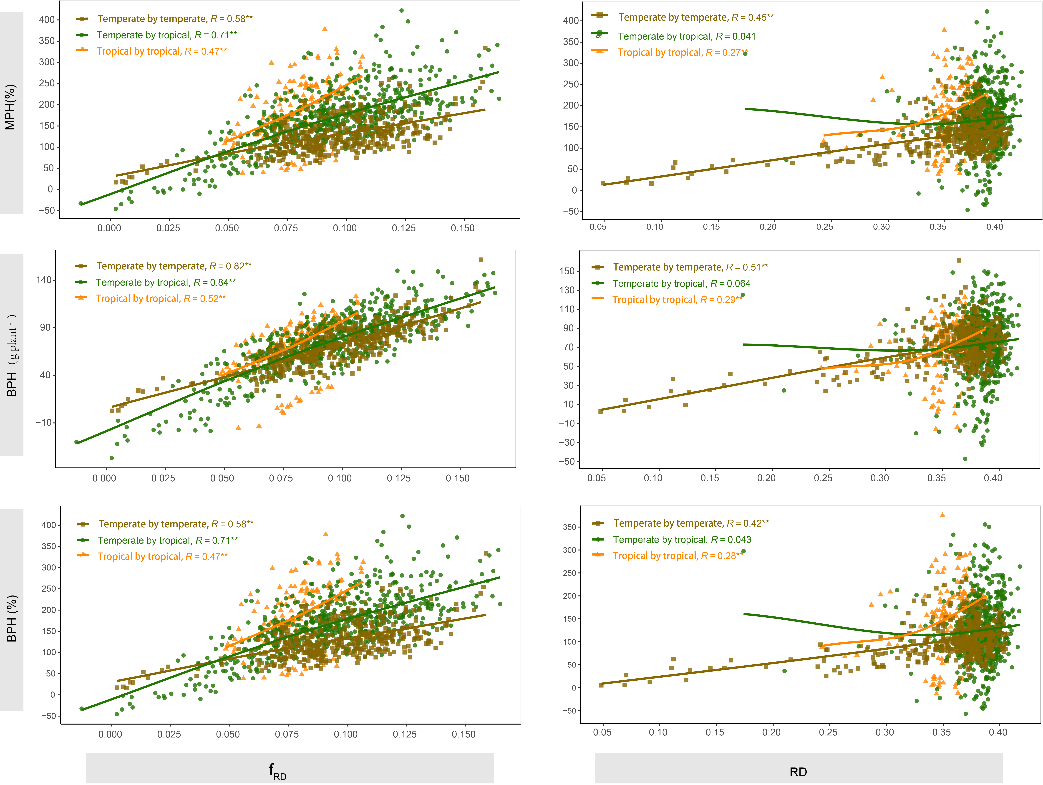


**Figure. S5 Association between grain weight per plant (GWPP) heterosis and genetic distance.** Association between relative mid-parent heterosis (MPH%), better-parent heterosis (BPH), and relative better-parent heterosis (BPH%) of GWPP and heterotic genetic distance ($\int_{\boldsymbol{RD}}$) or Rogers’ distance (RD). The colored trendlines are locally weighted regression lines for the temperate by temperate (golden), temperate by tropical (green), and tropical by tropical (orange).


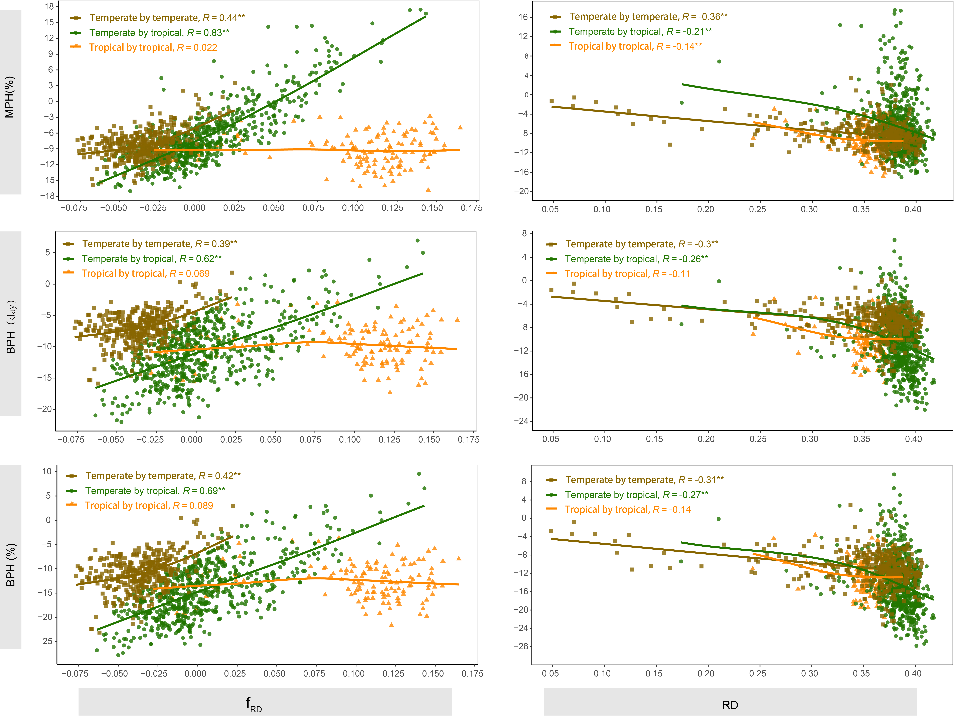


**Figure. S6 Association between days to silking (DTS) heterosis and genetic distance.** Association between relative mid-parent heterosis (MPH%), better-parent heterosis (BPH), and relative better-parent heterosis (BPH%) of DTS and heterotic genetic distance ($\int_{\boldsymbol{RD}}$) or Rogers’ distance (RD), shown for the different sets of hybrids. The colored trendlines are locally weighted regression lines.


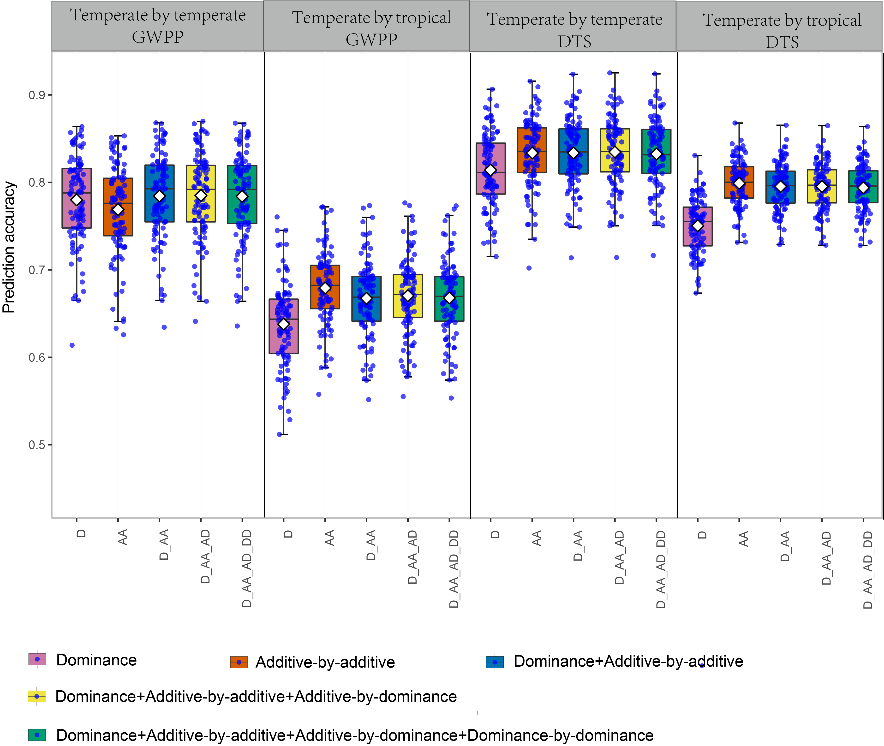


**Figure. S7 Genome-wide prediction accuracies for mid-parent heterosis in the temperate by temperate and temperate by tropical sets considering different genetic effects with five-fold cross-validation for 100 iterations.** GWPP, Grain weight per plant; DTS, Days to silking.


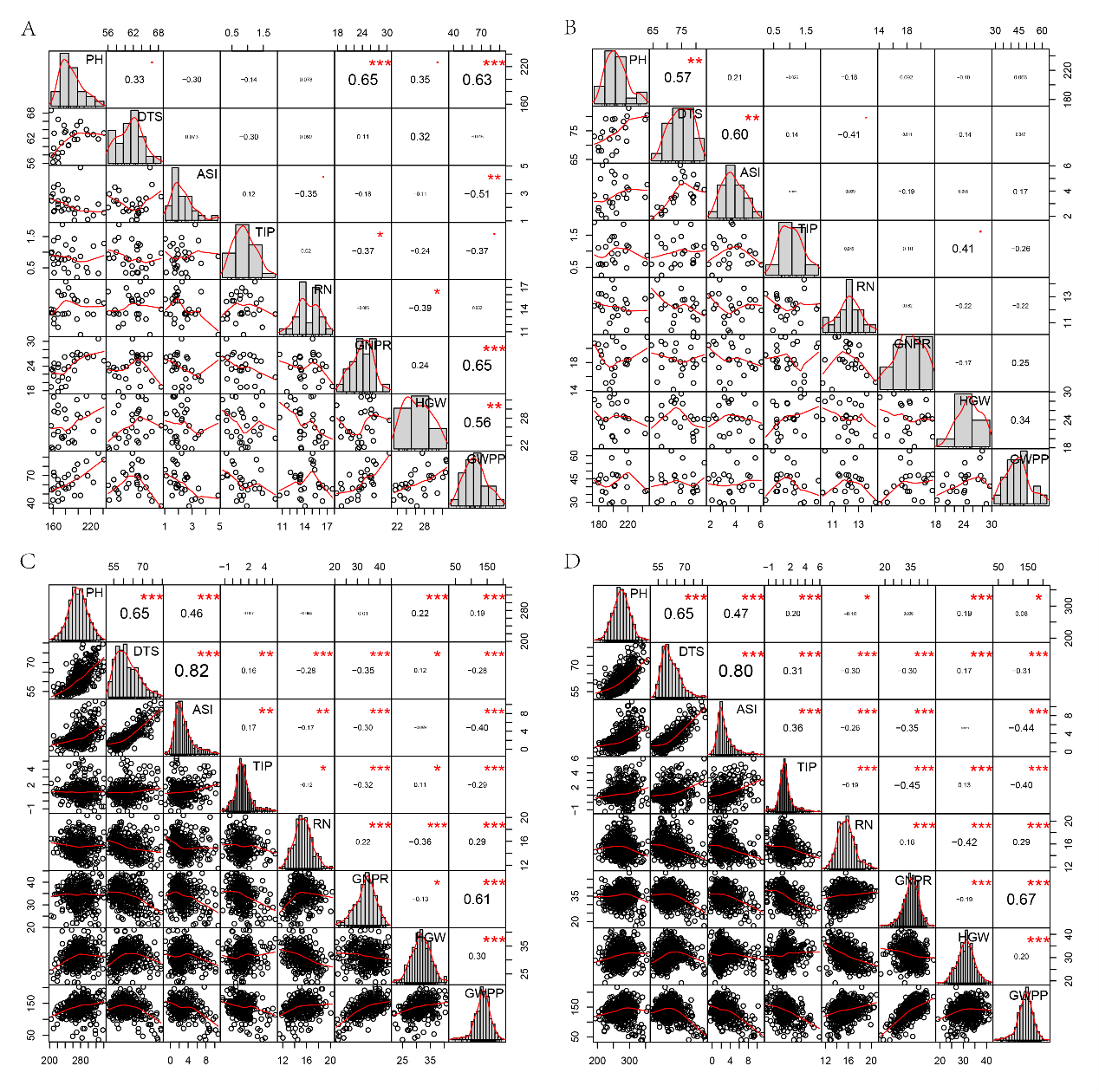
**Figure. S8 Phenotypic trait correlations.** (A) temperate lines and (B) tropical lines. (C) Temperate by temperate hybrids and (D) temperate by tropical hybrids. Grain weight per plant (GWPP) was found to be a weakly positive correlated with plant height (PH), grain number per row (GNPP), and hundred-grain weight (HGW) within the tropical lines. In contrast, for the hybrids of the temperate parental lines strongly positive correlations were observed. DTS, Days to silking; ASI, Anthesis-silking interval; TIP, Bare tip; RN, Row number. ***, **, and *indicate significance at 0.001, 0.01, and 0.05 levels, respectively.


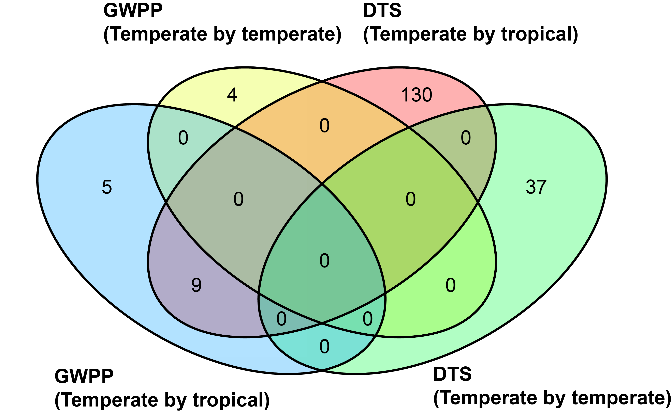


**Figure. S9** **Venn diagram showing the number of intersecting heterotic QTLs between the temperate by tropical hybrids and temperate by temperate hybrids for grain weight per plant (GWPP) and days to silking (DTS).**


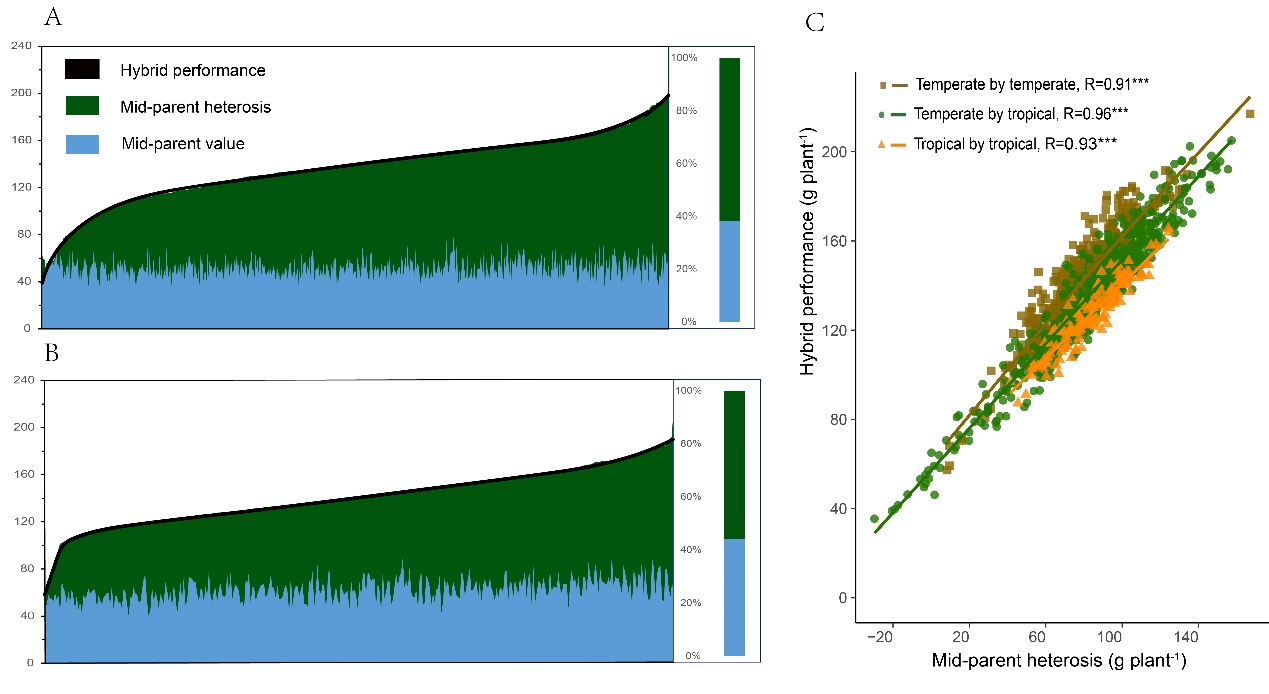


**Figure. S10 Mid-parent heterosis and hybrid performance for grain yield.** (**A**) Hybrids of the temperate by tropical set ordered for increasing hybrid performance, illustrating the contribution of mid-parent value and heterosis. Average mid-parent value and heterosis in the temperate by tropical set. Mid-parent heterosis contributed to 61.5% of the variance of the hybrid performance. (**B**) Hybrids of the temperate by temperate set were ordered for increasing hybrid performance, and average mid-parent value and heterosis. Mid-parent heterosis contributed to 55.7% of the variance of the hybrid performance. (**C**) A positive association between mid-parent heterosis and hybrid performance.

**Table S1. Location information.** Locations used for the field trials in the growing-season

| Location | Latitude | Longitude | Altitude | Mean annual precipitation | Set(amount) | year |
| --- | --- | --- | --- | --- | --- | --- |
| Xinxiang | 113°80 E | 35°16 N | 75 m | 573.4 mm | TT (325); TP (263) | 2013-2015 |
| Shunyi | 116°46 E | 40°23 N | 35 m | 610.0 mm | TT (325); TP (263) | 2013-2015 |
| Hongjing | 100°82 E | 22°03 N | 552 m | 1139.3 mm | PP (136) | 2014 |
| Sanya | 109°23 E | 18°43 N | 9 m | 1347.5 mm | PP (136) | 2015 |
| Shihezi | 44°51 E | 85°23 N | 450 m | 186.2 mm | TT (377); TP (641) | 2017-2018 |

TT, temperate by temperate; TP, temperate by tropical; PP, tropical by tropical.

**Table S2.** Correlations among marker-derived kinship matrices of dominance (D), and respective digenic epistatic (AA, AD, DD) effects in the genome-wide prediction model partitioning genetic variance components within the temperate by tropical set

|  | D | AA | AD |
| --- | --- | --- | --- |
| AA | 0.5622 |  |  |
| AD | 0.8904 | 0.8632 |  |
| DD | 0.8263 | 0.8395 | 0.9657 |

**Table S3.** Correlations among marker-derived kinship matrices of dominance (D), and respective digenic epistatic (AA, AD, DD) effects in the genome-wide prediction model partitioning genetic variance components within the temperate by temperate set

|  | D | AA | AD |
| --- | --- | --- | --- |
| AA | 0.6514 |  |  |
| AD | 0.8834 | 0.9103 |  |
| DD | 0.87 | 0.8611 | 0.9498 |

**Table S4.** The estimated variance components (var) and proportion of genetic variance (Pg) for mid-parent heterosis of grain weight per plant and days to silking which can be predicted using genomic prediction models exploiting different genetic effects with five-fold cross-validation for 100 iterations.

| Trait | Set | varD | varAA | varAD | varDD | varE | Pg |
| --- | --- | --- | --- | --- | --- | --- | --- |
| GWPP | TP | 143.4 | 77.1 | 91.3 | 67.4 | 174.9 | 0.6843 |
|  | TT | 108.9 | 45.3 | 50.8 | 45.4 | 68.3 | 0.7856 |
| DTS | TP | 0.91 | 1.65 | 1.17 | 0.67 | 2.28 | 0.6593 |
|  | TT | 0.59 | 0.34 | 0.33 | 0.23 | 0.35 | 0.8076 |

TT, temperate by temperate; TP, temperate by tropical; D, Dominance; AA, Additive-by-additive; AD, Additive-by-dominance; DD, Dominance-by-dominance; GWPP, Grain yield per plant; DTS, Days to silking.
